# Examination of Factors Affecting Site-Directed RNA Editing by the MS2-ADAR1 Deaminase System

**DOI:** 10.3390/genes14081584

**Published:** 2023-08-04

**Authors:** Md Thoufic Anam Azad, Umme Qulsum, Toshifumi Tsukahara

**Affiliations:** 1 Area of Bioscience, Biotechnology and Biomedical Engineering Research Area, Japan Advanced Institute of Science and Technology (JAIST), 1-1 Asahidai, Nomi City 923-1292, Ishikawa, Japan; thoufic@ru.ac.bd (M.T.A.A.);; 2 Department of Veterinary and Animal Sciences, University of Rajshahi, Rajshahi 6205, Bangladesh; 3 Department of Botany, Faculty of Biological Sciences, University of Rajshahi, Rajshahi 6205, Bangladesh; 4 GeCoRT Co., Ltd., 2-11-2 Takashima, Nishi-ku, Yokohama 220-0011, Kanagawa, Japan

**Keywords:** efficiency, gene therapy, RNA editing

## Abstract

Adenosine deaminases acting on RNA (ADARs) have double-stranded RNA binding domains and a deaminase domain (DD). We used the MS2 system and specific guide RNAs to direct ADAR1-DD to target adenosines in the mRNA encoding-enhanced green fluorescence protein. Using this system in transfected HEK-293 cells, we evaluated the effects of changing the length and position of the guide RNA on the efficiency of conversion of amber (TAG) and ochre (TAA) stop codons to tryptophan (TGG) in the target. Guide RNAs of 19, 21 and 23 nt were positioned upstream and downstream of the MS2-RNA, providing a total of six guide RNAs. The upstream guide RNAs were more functionally effective than the downstream guide RNAs, with the following hierarchy of efficiency: 21 nt > 23 nt > 19 nt. The highest editing efficiency was 16.6%. Off-target editing was not detected in the guide RNA complementary region but was detected 50 nt downstream of the target. The editing efficiency was proportional to the amount of transfected deaminase but inversely proportional to the amount of the transfected guide RNA. Our results suggest that specific RNA editing requires precise optimization of the ratio of enzyme, guide RNA, and target RNA.

## 1. Introduction

Genome editing has gained considerable attention in recent years, especially since the development of the CRISPR-Cas9 system [1]. Although this system is a powerful editing technique and genome editing for therapeutic purposes has great potential, its use is complicated by ethical issues and the possibility of genomic mutations that might have long-term adverse effects on the individual or their progeny [2]. RNA editing is also attracting growing interest [3] and is more promising than genome editing in terms of safety, ethical considerations, and choice [4,5,6]. Therefore, researchers have been focusing on the development of improved RNA editing approaches [7,8].

The conversion of adenosine (A) to inosine (I), which is read as guanosine (G) during translation, is induced by adenosine deaminase acting on RNA (ADAR) and is the most common form of RNA editing in mammals. ADARs harbor double-stranded RNA binding domains and a deaminase domain (DD). The majority of RNA editing events occur in the central nervous system [9], and altered RNA editing is associated with several diseases related to the neuromuscular system. A-to-I RNA editing can alter 12 of the 20 canonical amino acids and can also recode the stop codons (UGA, UAG, and UAA) to tryptophan (UGG) [10]. This technique is utilized to correct a wide variety of disease-related mutations, such as those associated with cystic fibrosis [11], Duchenne muscular dystrophy [12], and Factor V Leiden thrombophilia [13].

The MS2 system is used widely as an RNA binding system [14]. Specifically, the bacteriophage MS2 protein binds to its corresponding MS2 stem-loop RNA, which can be attached to guide RNAs that target specific sequences. In our previous study, we genetically engineered a chimeric MS2-ADAR1-DD protein capable of performing specific A-to-I conversions and used it alongside guide RNAs that directed the deaminase to the desired editing site in transiently transfected HEK-293 cells [15]. Using this system, we reported an RNA editing efficiency of approximately 7% for conversion of the amber stop codon (TAG) of enhanced green fluorescent protein (*EGFP*) to tryptophan (TGG), with no evidence of off-target editing [15]. In subsequent studies, we evaluated the RNA editing efficiencies of ADAR variants [16] and upgraded the efficiency of the editing system via modification of the effector molecules [17].

Balancing high-editing efficiency with a low rate of off-target effects is a major issue when considering therapeutic applications of RNA editing [18]. Several factors can affect the efficiency of A-to-I RNA editing, such as the neighboring sequences of the targeted nucleotide [19]. In addition, the length of the guide RNA is crucial for the efficacy and specificity of editing. In the current study, we examined the effects of different lengths and locations of guide RNAs, as well as those of different amounts of RNA editing factors, on RNA editing efficiency and off-target editing of the mutated *EGFP* mRNA. Guide RNAs of 19, 21, or 23 nt were positioned upstream and downstream of the MS2-RNA. The highest editing efficiency was 16.6%; however, one off-target editing site was detected in the *EGFP* mRNA and some editing of the target site occurred in the absence of a guide RNA. Our findings indicate that specific RNA editing requires precise optimization of the levels of deaminase enzyme, guide RNA, and target RNA. In addition, optimization of the promoters in the expression constructs and the tagged peptides adjacent to the MS2-ADAR1-DD might influence the functionality of this system for therapeutic applications.

## 2. Materials and Methods

### 2.1. MS2-ADAR1-DD Construct and Reporter Mutated EGFP Target Sequences

The MS2-ADAR1-DD construct used here was described in our previous studies [15,16]. The sequence of ADAR1-DD was checked against the UCSC Human Genome Browser [20]. The target sequence was the amber stop codon (TAG) at nucleotide position 58 of the EGFP mRNA. To examine the editing site preference of MS2-ADAR1-DD, the efficiency of double A-to-I editing of the ochre stop codon (TAA) was also examined.

### 2.2. Preparation of Guide RNA Constructs

We designed 19, 21, and 23 nt guide RNAs that were complementary to the target RNA and positioned them upstream or downstream of the MS2-RNA (Table 1). The MS2 RNA sequence was amplified using the following primers: MS2RNARv, ATTCCTCGAGCGCAAATTTAAAGCGCTGAT; MS2RNAFw, GATTACGAATTCGAATGGCCATG. Each guide sequence was inserted into the pCS2+ plasmid (Addgene) at the EcoRI and XhoI restriction sites [15]. Briefly, the pCS2+ plasmid and a PCR product comprising the MS2-RNA (6×) and guide RNA sequence were digested with EcoRI and Xhol (Takara, Shiga, Japan) in separate reactions. The digested vector was incubated for 30 min with bovine alkaline phosphatase, followed by ethanol precipitation. The purified products were separated by 1% agarose gel electrophoresis and the desired bands were extracted during a short exposure to UV transillumination. A Qiagen gel extraction kit (Qiagen, Hilden, Germany) was used to purify the vector and insert. Subsequently, ligation was performed using Mighty Mix (Takara), according to the manufacturer’s instructions.

### 2.3. Cell Culture and Transfection

HEK-293 cells were seeded into 24-well plates (Costar, Corning, NY, USA) at a density of 1.5 × 10^5^ cells/well. After incubation overnight at 37 °C and 5% CO_2_, the cells were transfected with varying amounts of the guide RNAs and deaminase using Lipofectamine 3000 (Invitrogen, Carlsbad, CA, USA), according to the manufacturer’s instructions. At 6 h post-transfection, the transfection medium was replaced with fresh DMEM (WAKO, Tokyo, Japan) containing 10% fetal bovine serum (GIBCO, Thermo Fisher Scientific, Waltham, MA, USA). The amount of transfected EGFP reporter (10 ng) was constant in each experiment.

### 2.4. RNA Extraction, cDNA Synthesis, and PCR

At 72 h post-transfection, RNA was extracted from cells using TRIzol reagent (Invitrogen). Briefly, the culture medium was aspirated, the cells were washed gently with 1 × PBS, and then TRIzol (500 µL) was added to each well and the cells were homogenized by pipetting. Subsequently, chloroform (200 µL) was added to the homogenates, followed by mixing and incubation at room temperature for 5 min. The tubes were then centrifuged at 15,000 rpm for 20 min at 4 °C. The clear aqueous phase was transferred to a tube containing 200 µL of ice-cold isopropanol and the samples were mixed by inverting the tubes several times. Subsequently, the samples were centrifuged as described above and the supernatant was discarded. The RNA pellet was washed with 70% ethanol and air dried. Next, the extracted RNA was treated with DNase (Promega, Fitchburg, WI, USA), according to the manufacturer’s instructions. Synthesis of cDNA was performed using EGFP-specific primers and Superscript III (Invitrogen), according to the manufacturer’s instructions. PCR amplification of the 324 bp product was performed using GoTaq polymerase (Promega), with annealing at 60 °C and extension at 72 °C for 30 s. A total of 40 PCR cycles were performed to generate the desired concentration of PCR product.

### 2.5. Purification of PCR Products, Sanger Sequencing, and Quantification of RNA Editing

PCR products were run on 6% polyacrylamide gels for 20 min in presence of 1 × TBE buffer. The required bands were excised from the gels under low-level, short UV transillumination. Subsequently, the gel pieces were frozen for 30 min at −80 °C and then crushed using a disposable pellet pestle/tissue grinder (Kimble^®^; Capitol Scientific, Inc., Austin, TX, USA; Catalog no. 749520-0090). Next, 0.1 × TE (10 µL) was added and the samples were vortexed for 10 min. After centrifugation for 10 min at high speed, the supernatant was collected for use in sequencing reactions. To assess the C/T peak height at the targeted adenosine, antisense sequencing was performed on a 3130xl genetic analyzer (Applied Biosystems, Foster City, CA, USA) using the reverse PCR primer. The efficiency of RNA editing (%) was calculated from the C/T peak height [21,22]. All values were calculated based on at least three independent experiments.

### 2.6. Statistical Analysis

All data were obtained from three independent replication of the experiments. Data were plotted in the .XL file. Data were presented as mean value ± SD for biological replicates; data were statistically compared using unpaired Student’s *t*-test. * Indicates *p* value < 0.05 and statistically significant and ** indicates *p* value < 0.01 is statistically more significant.

## 3. Results

### 3.1. Effects of the Length and Position of Guide RNAs on Their Editing Efficiency

**Figure 1:**
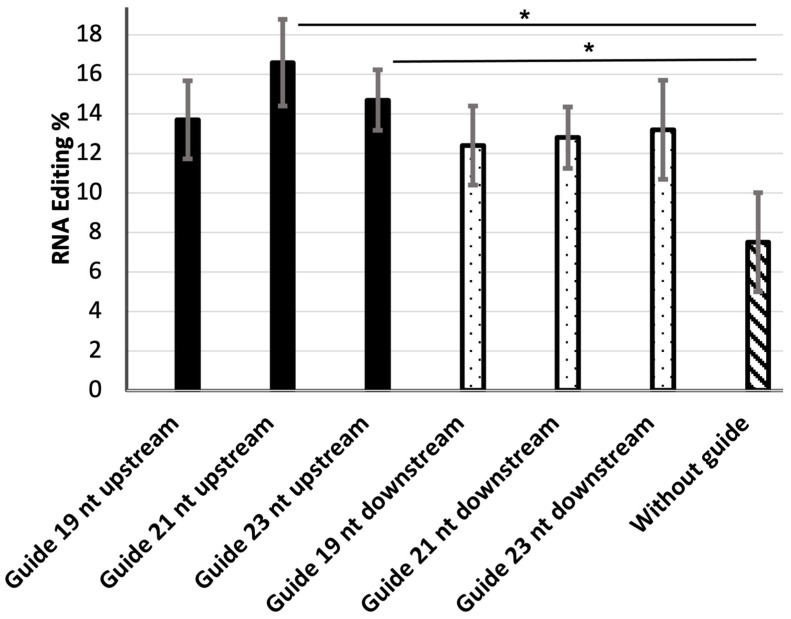
The editing efficiencies of the six guide RNAs, located upstream or downstream of MS2-RNA. * Indicates *p* value < 0.05 and statistically significant.

Guide RNAs of 19, 21, and 23 nt, complementary to the target site, were positioned either upstream or downstream of the MS2-RNA, providing a total of six guide RNAs. Editing efficiency was examined in HEK-293 cells transfected with a guide RNA construct, an EGFP construct, and the MS2-ADAR1-DD construct. The A-to-I editing efficiency was determined by Sanger sequencing to assess the C/T peak height at the targeted adenosine in the amber stop codon of EGFP (TAG). Overall, the editing efficiency was higher for upstream guide RNAs than for their downstream equivalents (Figure 1).

For the upstream guide RNAs, the editing efficiency was highest (16.6%) for the 21 nt guide RNA and lowest (12.7%) for the 19 nt upstream guide RNA. For the downstream guide RNAs, the editing efficiency was highest (13.2%) for the 23 nt guide RNA and lowest (12.4%) for the 19 nt guide RNA. In the absence of a guide RNA, the efficiency of target sequence editing was 7.5% (Figure 1 and Figure S1).

### 3.2. Effect of the Amount of Guide RNA on Editing Efficiency

Next, we examined the effect of varying the amount of transfected guide RNA on target editing efficiency. HEK-293 cells were transfected with 500 ng of MS2-ADAR1-DD and 250, 500, or 1000 ng of the 21 nt upstream guide RNA. The editing efficiency was highest (12.9%) for 250 ng and lowest (4.4%) for 1000 ng of guide RNA.

**Figure 2:**
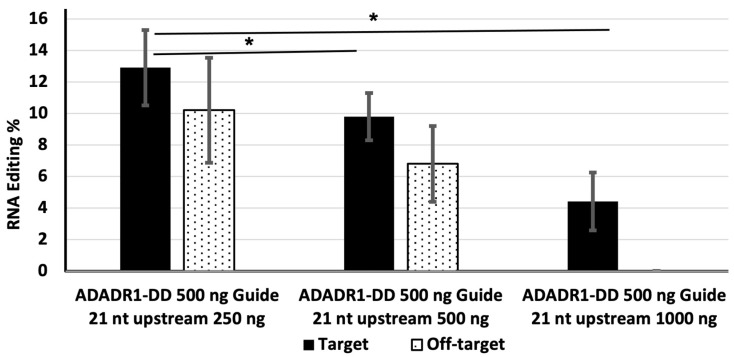
The effect of the amount of guide RNA on the efficiency of target and off-target RNA editing. HEK-293 cells were transfected with 500 ng of MS2-ADADR1-DD and 250, 500, or 1000 ng of the 21 nt upstream guide RNA. The solid bar indicates editing of the target site and dotted bars indicate off-target editing. * Indicates *p* value < 0.05 and statistically significant.

Off-target editing was also detected in cells transfected with 250 and 500 ng of guide RNA (6.8% and 10.2%, respectively), although it was not detected in cells transfected with 1000 ng of guide RNA (Figure 2).

### 3.3. Effect of the Amount of MS2-ADAR1-DD on Editing Efficiency

**Figure 3:**
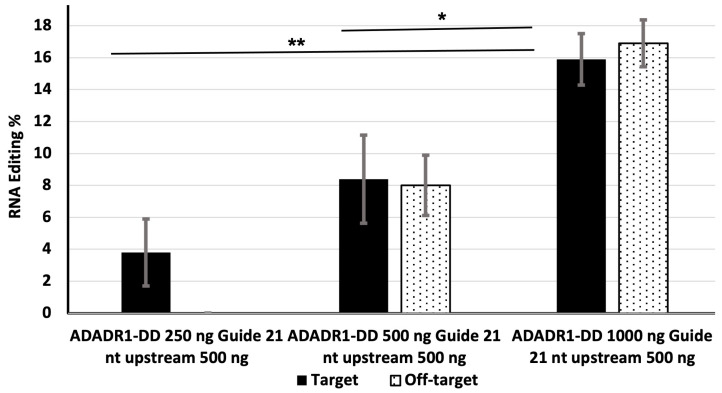
The effect of the amount of MS2-ADAR1-DD on the efficiency of target and off-target RNA editing. HEK-293 cells were transfected with 500 ng of the 21 nt upstream guide RNA and 250, 500, or 1000 ng of MS2-ADADR1-DD. The solid bar indicates editing of the target site and dotted bars indicate off-target editing. * Indicates *p* value < 0.05 and statistically significant and ** indicates *p* value < 0.01 is statistically more significant.

We also examined the effect of varying the amount of deaminase on editing efficiency. HEK-293 cells were transfected with 500 ng of the 21 nt upstream guide RNA and 250, 500, or 1000 ng of MS2-ADAR1-DD. The editing efficiencies at 250, 500, and 1000 ng of deaminase were 3.8%, 8.4%, and 15.9%, respectively (Figure 3).

At 500 and 1000 ng, off-target editing was detected 50 nt downstream of the target, with efficiencies of approximately 8.0% and 16.9%. By contrast, off-target editing was almost undetectable in cells transfected with 250 ng of deaminase (Figure 3 and Figure S2).

### 3.4. Effect of the Amounts of MS2-ADAR1-DD and Guide RNA on Editing Efficiency in Cells Expressing a Double Repeated 19 nt 2× nt Upstream Guide RNA

**Figure 4:**
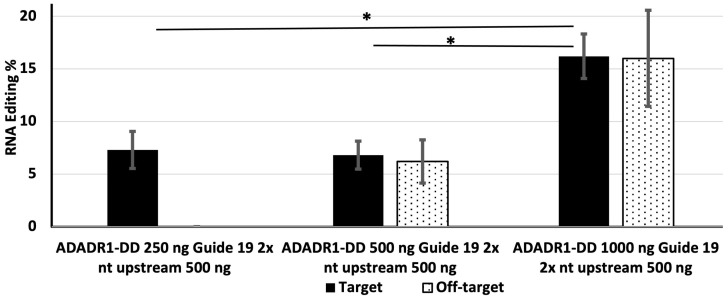
The effect of the amount of MS2-ADADR1-DD on the efficiency of target and off-target editing in cells expressing a double repeated 19 nt 2× upstream guide RNA. HEK-293 cells were transfected with 500 ng of the guide RNA and 250, 500, or 1000 ng of MS2-ADADR1-DD. The solid bar indicates editing of the target site and dotted bars indicate off-target editing. * Indicates *p* value < 0.05 and statistically significant.

Next, we examined the editing efficiencies of different amounts of MS2-ADAR1-DD in the presence of 500 ng of a double-repeated 19 nt 2× upstream guide RNA. Cells were transfected with the EGFP construct, 500 ng of the double repeated guide RNA, and 250, 500, or 1000 ng of MS2-ADAR1-DD. In this case, the target editing efficiency was highest (16.2%) for 1000 ng of MS2-ADADR1-DD, whereas 250 ng of MS2-ADADR1-DD yielded an editing efficiency of 7.3% (Figure 4). Off-target editing was also high (16%) for 1000 ng of MS2-ADADR1-DD, but was undetectable in cells transfected with 250 ng of the deaminase (Figure 4).

**Figure 5:**
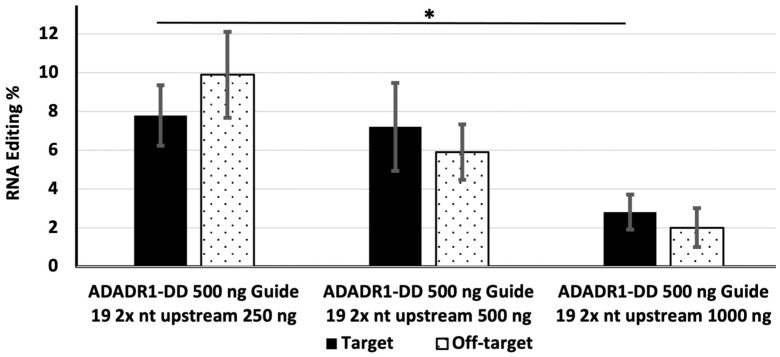
The effect of the amount of double repeated 19 nt 2× upstream guide RNA on the efficiency of target and off-target editing. HEK-293 cells were transfected with 500 ng of MS2-ADADR1-DD and 250, 500, or 1000 ng of the guide RNA. The solid bar indicates editing of the target site and dotted bars indicate off-target editing. * Indicates *p* value < 0.05 and statistically significant.

Subsequently, we kept the amount of deaminase constant at 500 ng and varied that of the double repeated 19 nt 2× upstream guide RNA (Figure 5).

The editing efficiency was highest (7.8%) at 250 ng of the guide RNA and lowest (2.8%) at 1000 ng of the guide RNA (Figure 5). Off-target editing (9.9%) was also detected at 250 ng of the guide RNA.

### 3.5. Efficiency and Site Preference of the MS2-ADAR1-DD System in Conversion of the Ochre Stop Codon (TAA) to Tryptophan (TGG)

**Figure 6:**
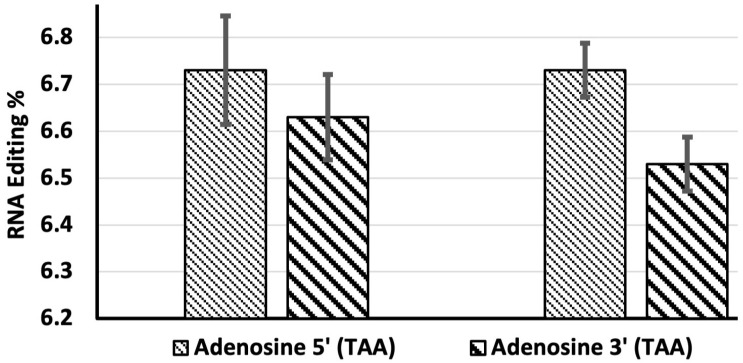
The efficiency of conversion of stop codon (TAA) to the tryptophan codon (TGG). HEK-293 cells were transfected with 500 ng of MS2-ADADR1-DD and 250 ng of the 21 nt upstream guide RNA. The efficiencies of editing of the 5′ (TAA) and 3′ (TAA) adenosines are shown. Replicates of the experimental data are presented side by side.

To determine the editing site preference of MS2-ADAR1-DD, the efficiency of double A-to-I editing of the ochre stop codon (TAA) of EGFP was examined. To this end, HEK-293 cells were transfected with 500 ng of MS2-ADAR1-DD and 250 ng of the 21 upstream guide RNA. We found that the 5′ adenosine (TAA) was edited with an efficiency of approximately 6.7%, whereas the 3′ adenosine (TAA) was edited with an efficiency of approximately 6.6% (Figure 6).

## 4. Discussion

In this study, we used guide RNAs that differed in length from 19 to 23 nt (Table 1) and examined their abilities to promote editing of the EGFP mRNA by the MS2-ADAR1-DD deaminase. We found that the editing efficiency of the 21 nt guide RNA was higher than those of the 19 and 23 nt guide RNAs (Figure 1). Regarding the position of the guide RNA, for all three lengths, those located upstream of MS2-RNA were more functionally efficient than those located downstream (Figure 1). It is possible that 19 nt guide RNAs bind less efficiently to the target site than 21 bp guide RNAs, whereas 23 nt guide RNAs may bind strongly and inhibit polymerase activity, thus reducing the editing efficiency. Notably, we observed a considerable level off-target site editing (7.5%) in the absence of guide RNA, which may have been due to the high concentrations of ADAR1 and EGFP mRNA in the cytoplasm, resulting in a close molecular interaction between these molecules.

Off-target conversion is a challenge for RNA editing. It can occur in the region complementary to the guide RNA or in another portion of the targeted RNA. One group reported that the rate of off-target editing can be higher than that of target site editing [23] complementarity of the guide RNA [11]. It can also be minimized by changing the concentration of guide RNA, although this can sometimes decrease the target site editing efficiency [23]. The addition of a nuclear localization signal sequence at the N-terminal region of the DD can also reduce off-target editing [17,23]. In our current study, off-target editing (TAC > TGC) was detected 50 nt downstream of the target site (TAG > TGG) and resulted in a missense mutation (Y > C) in the EGFP sequence (Figure S3). Although off-target editing was not detected when cells were transfected with 250 ng of MS2-ADAR1-DD, it was present in cells transfected with 500 or 1000 ng of MS2-ADAR1-DD, and its efficiency was proportional to the deaminase level (Figure 3 and Figure 4). By contrast, a previous study found that off-target editing was related to the concentration of guide RNA [24].

Although several probable off-target (TAC) sites were located within the region surrounding the target site in our current analysis (Figure S3), Sanger sequencing of approximately 50% of the EGFP transcript length revealed only one off-target editing event, located 50 nt downstream of the targeted adenosine. Notably, there was a high GC content in the regions adjacent to the target and off-target adenosines (Figure S3). It is possible that off-target editing was mediated by the presence of free deaminase in the cytoplasm and its prolonged association with off-target sites in the EGFP mRNA. Alternatively, it may have been attributable to the complex secondary structure of the mutated EGFP mRNA, which led to contact of the off-target editing site with the deaminase grove. Here, no off-target editing was detected in the complementary regions of the guide RNAs (Figures S2 and S4–S6). Multiple off-target editing sites within the region complementary to the guide RNA sequence were reported for ADAR2-DD and ADAR2-DD (E488Q) [23], and most previous studies reported that off-target editing occurs at nucleotides adjacent to the target [25]. It can be crucial to eliminate or minimize such off-target conversions; however, although off-target effects are undesirable, it is worth noting that RNA editing is a transient process and does not affect the genome. Hence, off-target RNA editing is not as malignant as off-target genome editing.

In the presence of 500 ng of the 21 nt upstream guide RNA, 250 ng MS2-ADADR1-DD resulted in a target editing efficiency of 3.8% (Figure 3). When the amount of MS2-ADAR1-DD was increased to 500 or 1000 ng, the editing efficiency increased proportionally to ~8% and ~16%, respectively (Figure 3), indicating a deaminase concentration-dependent effect. However, as mentioned above, off-target editing also increased as the deaminase level increased. Therefore, fine-tuning the concentration of the deaminase in an RNA editing system is critical to obtain optimum genetic correction with minimum off-target editing. Endogenous integration of an RNA editing system can increase the editing efficiency [4,25,26,27]; however, this advantage comes at the cost of limited control of the endogenous promoter directing expression of the deaminase. This limitation can be minimized by transient transfection and optimizing the effector molecule concentration. Indeed, a recent CRISPR-meditated approach to cystic fibrosis therapy approach was based on transient application of a modified CRISPR system to edit the gene encoding cystic fibrosis transmembrane conductance regulator [28]. dCas13-ADAR fusions for site directed mutagenesis is a powerful technique with higher RNA editing efficiency [7]. Unfortunately, off-target editing is much higher in this case [7,8]. ADAR enzymes naturally modified to bind to the double stranded RNA. Integration of the Cas13 with ADARs increase the molecular weight and cargo size for the gene delivery system. In our MS2-ADARs-DD system [15,16,17] the modification is less than that and the programming is straight forward. Although editing efficiency lower than the dCas13-ADAR [7,8] to some extend but still there are many points to address and tuning for higher editing efficacy [17] and specificity. However, we cannot exclude the possibility of increased number of off-target in MS2-ADAR1-DD system when screening to genome-wide.

We found that when we kept the deaminase level constant at 500 ng, there was an inverse relationship between the guide RNA concentration and both the target and off-target editing efficiencies (Figure 2). This finding may have been due to increased binding of high amounts of the chimeric deaminase to the guide RNA, which would limit its availability for editing of the EGFP RNA. Alternatively, it may have been due to competitive transfection of the plasmids, whereby high amounts of the guide RNA plasmid limited the amount of deaminase plasmid transfected into cells.

In addition to examining the effects of guide RNA length on RNA editing efficiency, we also investigated the editing efficiency in cells transfected with a plasmid expressing a double-repeated 19 nt 2× upstream guide RNA. When 500 ng of the double-repeated 19 nt guide RNA was used alongside 250 ng of deaminase, the target editing efficiency was >7.8% (Figure 4) and it is higher than the 21 bp upstream guide (Figure 2). When the amount of deaminase was increased from 250 ng to 500 ng in cells expressing the double repeated 19 nt guide RNA, the editing efficiency was not increased. However, when the amount of deaminase was increased to 1000 ng, the editing efficiency was increased to approximately 16.2% (Figure 4), a level that was similar to the editing efficiency in cells expressing the 21 nt single upstream guide RNA (16.7%; Figure 1). By contrast, increasing the amount of the double-repeated 19 nt 2× upstream guide RNA while maintaining a constant amount of deaminase reduced the editing efficiency (Figure 5). A previous study reported that the use of a repeated guide λN-RNA led to increased editing efficiency [29]; however, in our current study, we did not observe increased editing efficiency in cells expressing the repeated guide RNA. Therefore, more detailed investigations are needed to explain this phenomenon. We cannot exclude the possibility of lack of proper positioning of the guide RNA or the inhibition of editing caused by deaminases interacting at adjacent locations on the target.

In our previous study, we used an allele of EGFP in which the 58th codon was mutated from TGG (encoding tryptophan) to an ochre stop codon (TAA), and confirmed that editing of TAA back to TGG switched on EGFP fluorescence in HEK-293 cells [15]. In our current study, we examined the preference of the deaminase for the 5′ and 3′ adenosines in the TAA codon. We found that the 5′ adenosine (TAA) was edited more efficiently than the 3′ adenosine (TAA) (Figure 6), suggesting a preference for the 5′ adenosine by the deaminase system. Off-target editing was not observed in the complementary regions of the guide RNA as well (Figure S7).

The wild-type DD of ADAR2 is more specific than the mutated ADAR2-DD (E488Q), but has a lower editing efficiency [19,23]. In our current study, we used ADAR1-DD, which we found to be more functional than ADAR2-DD [16]. When used with λN-RNA in frog oocytes, the editing efficiency of ADAR2-DD was approximately 20% when the same technique was used in HEK-293 cells, the efficiency dropped to approximately 12% [11]. This difference may have been due to those associated with the techniques used to insert the effector molecules into cells [11]. Specifically, transfection is the most widely used method for gene delivery for cultured cells, whereas microinjection is typically used for eggs and zygotes. Unlike microinjection, transfection does not ensure that all reactive molecules are inserted simultaneously into the cell cytoplasm. Here, we found that the editing efficiency of ADAR1-DD in HEK-293 cells was up to ~16% and off-target editing was comparatively lower than that described previously. We tested this system in HeLa cells and observed the cell growth, confluency and efficacy of this RNA editing system. Except the green fluorescence signal, the microscopic images did not show any remarkable observable differences among the treatment and control groups of cells (Figure S8). Approximately 15% RNA editing is sufficient to restore a wild-type transcript [30].

## 5. Conclusions

RNA editing has promising prospects for gene therapy as it is efficient and has minimal side effects. Optimization of the concentrations of the effector molecules, including the guide RNA and enzymes, is needed for efficient RNA editing. In addition, fine-tuning of effector molecules is required to control off-target editing. Findings of this article shed light on the further optimization of in vivo approaches in mice model for the therapeutic purpose of human disease model as well. Gene delivery by the adenoviral vector can be utilized to deliver these editing factor at tissue level.

## Figures and Tables

**Table 1:** Sequences of the guide RNAs located upstream or downstream of MS2-RNA (6×). The underlined region indicates the guide sequence, and the bold underlined regions indicate the editing targets (CCA and TGG) and C/G mismatches to avoid off-target editing.

No.	Name	Restriction Enzyme Site, Guide with Primer Sequence (5′–3′)
1	Guide 19 bp upstream	ATCAGAATTCAGGGGGGG**CCA**GGGCACGGGAATGGCCATGGGACGTCGAC
2	Guide 21 bp upstream	ATCAGAATTCGAGGGGGGG**CCA**GGGCACGGGGAATGGCCATGGGACGTC
3	Guide 23 bp upstream	ATCAGAATTCCGAGGGGGGG**CCA**GGGCACGGGCGAATGGCCATGGGACGT
4	Guide 19 bp downstream	ATTCCTCGAGCCGTGCCC**TGG**CCCCCCCTCGCAAATTTAAAGCGCTGAT
5	Guide 21 bp downstream	ATTCCTCGAGCCCGTGCCC**TGG**CCCCCCCTCCGCAAATTTAAAGCGCTG
6	Guide 23 bp downstream	ATTCCTCGAGGCCCGTGCCC**TGG**CCCCCCCTCGCGCAAATTTAAAGCGCT
7	Guide 19 2 × upstream	ATCAGAATTCAGGGGGGG**CCA**GGGCACGGAGGGGGGG**CCA**GGGCACGGGAATGGCCATGGGACGTCGAC

## Data Availability

Not applicable.

## Supplementary Materials

The following supporting information can be downloaded at: https://www.mdpi.com/article/10.3390/genes14081584/s1, Figure S1: Sequencing results showing the comparative efficiencies of the six guide RNAs for EGFP RNA editing. Figure S2: Sequencing analysis of EGFP for cells transfected with 500 ng of 21 bp upstream guide RNA and 250 ng (A), 500 ng (B), or 1000 ng (C) of MS2-ADADR1-DD. Figure S3: The sequence of the mutated (A) and restored (B) EGFP site. The target site (TAG) is indicated. Off-target editing of TAC to TGC is also shown. Figure S4: Sequencing analysis of EGFP in cells transfected with 500 ng of MS2-ADADR1-DD and 250 ng (A), 500 ng (B), or 1000 ng (C) of 21 nt upstream guide RNA. Figure S5: Sanger sequencing analysis of EGFP in cells transfected with 500 ng of double repeated 19 nt 2× upstream guide RNA and 250 ng (A), 500 ng (B), or 1000 ng (C) of MS2-ADAR1-DD. Figure S6: Sequencing analysis of EGFP in cells transfected with 500 ng of MS2-ADADR1-DD and 250 ng (A), 500 ng (B), or 1000 ng (C) of double repeated 19 nt 2× upstream guide RNA. Figure S7: Sequencing analysis of the conversion of the ochre stop codon (TAA) to the tryptophan codon (TGG). (A) and (B) are replicates of the experiment. HEK-293 cells were transfected with 500 ng of MS2-ADADR1-DD and 250 ng of the 21 nt upstream guide RNA. Figure S8: Transfection experiment in HeLa cells. Bright image in left panel and fluorescence image in right panel of the same focus. Figures were taken by Keyence Biozero fluorescence microscope, BZ-X800.
