# Supplementary material for: Examination of Factors Affecting Site-Directed RNA Editing by the MS2-ADAR1 Deaminase System
Source: Genes (Basel). 2023 Aug 4;14(8):1584. doi: 10.3390/genes14081584 (PMC10454654; doi:10.3390/genes14081584)
Supplement: Supplementary file 1 [file genes-14-01584-s001.zip › genes-2530020-supplementary.pdf]

Examination of Factors Affecting Site-Directed RNA Editing by the MS2-ADAR1 Deaminase System

Md Thoufic Anam Azad [1,2,†], Umme Qulsum [1,3,†] and Toshifumi Tsukahara [1,4,*]

[1] Area of Bioscience, Biotechnology and Biomedical Engineering Research Area, Japan Advanced Institute of Science and Technology (JAIST), 1-1 Asahidai, Nomi City 923-1292, Ishikawa, Japan; thoufic@ru.ac.bd (M.T.A.A.); qulsum@ru.ac.bd (U.Q.)
[2] Department of Veterinary and Animal Sciences, University of Rajshahi, Rajshahi 6205, Bangladesh
[3] Department of Botany, Faculty of Biological Sciences, University of Rajshahi, Rajshahi 6205, Bangladesh
[4] GeCoRT Co., Ltd., 2-11-2 Takashima, Nishi-ku, Yokohama 220-0011, Kanagawa, Japan
* Correspondence: tukahara@jaist.ac.jp.
† Current address: Graduate School of Agricultural Science, Tohoku University, 232-3 Yomogida, Naruko-onsen, Osaki City 989-6711, Miyagi, Japan.

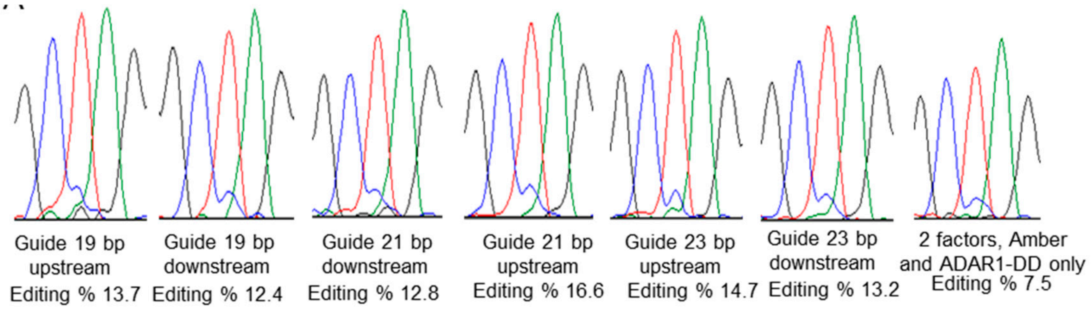

Supporting Figure S1. Sequencing results showing the comparative efficiencies of the six guide RNAs for *EGFP* RNA editing.

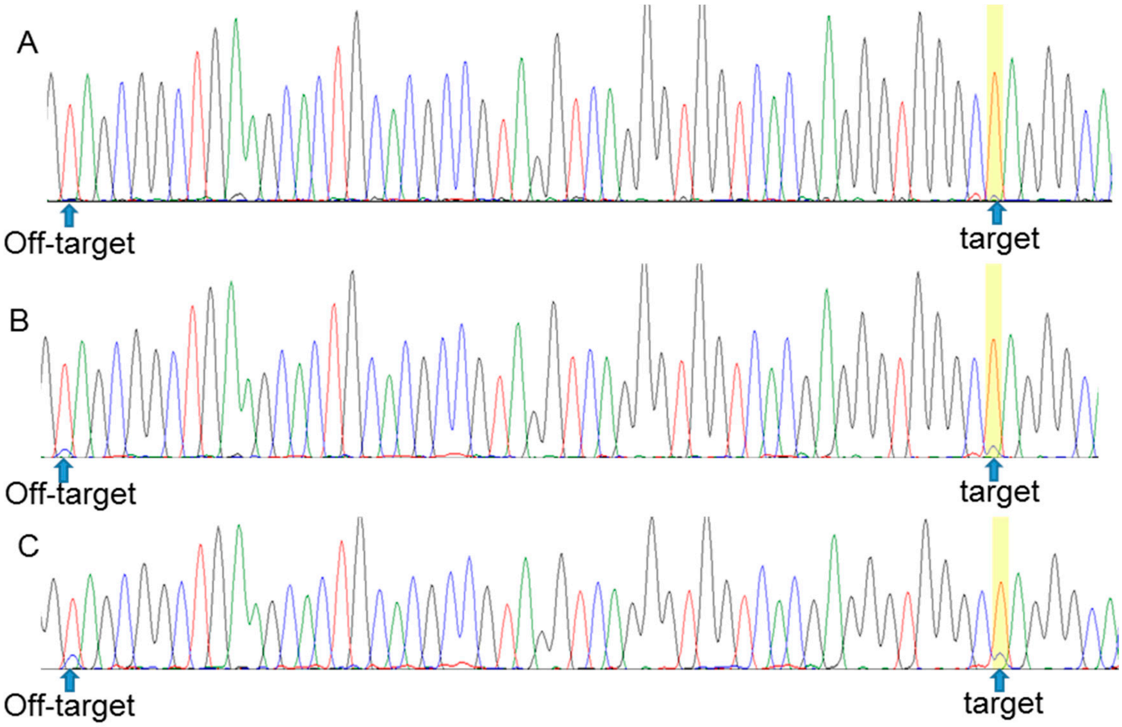

Supporting Figure S2. Sequencing analysis of *EGFP* for cells transfected with 500 ng of 21 bp upstream guide RNA and 250 ng (**A**), 500 ng (**B**), or 1000 ng (**C**) of MS2-ADADR1-DD.

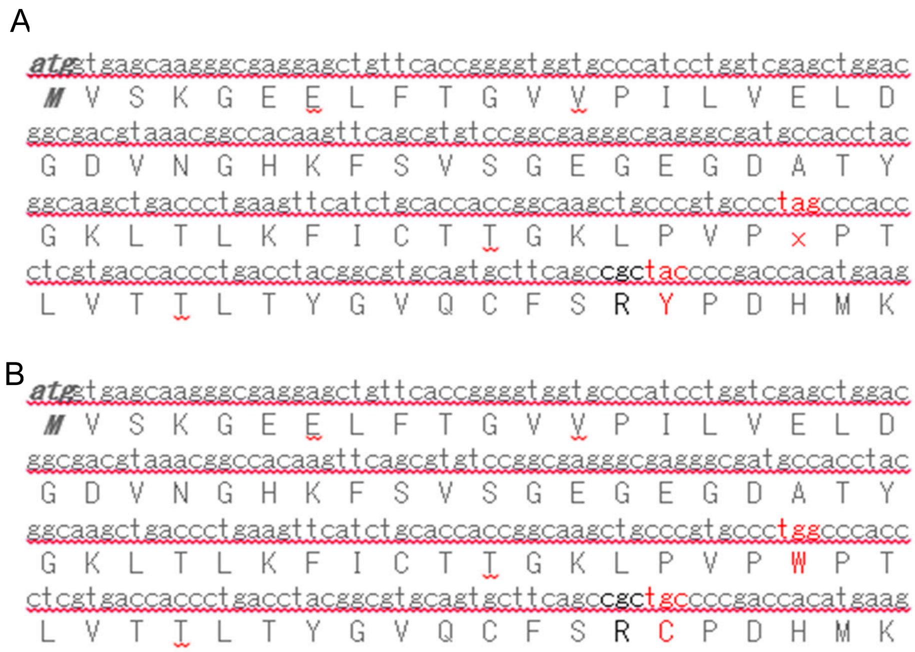

Supporting Figure S3. The sequence of the mutated (**A**) and restored (**B**) *EGFP* site. The target site (TAG) is indicated. Off-target editing of TAC to TGC is also shown.

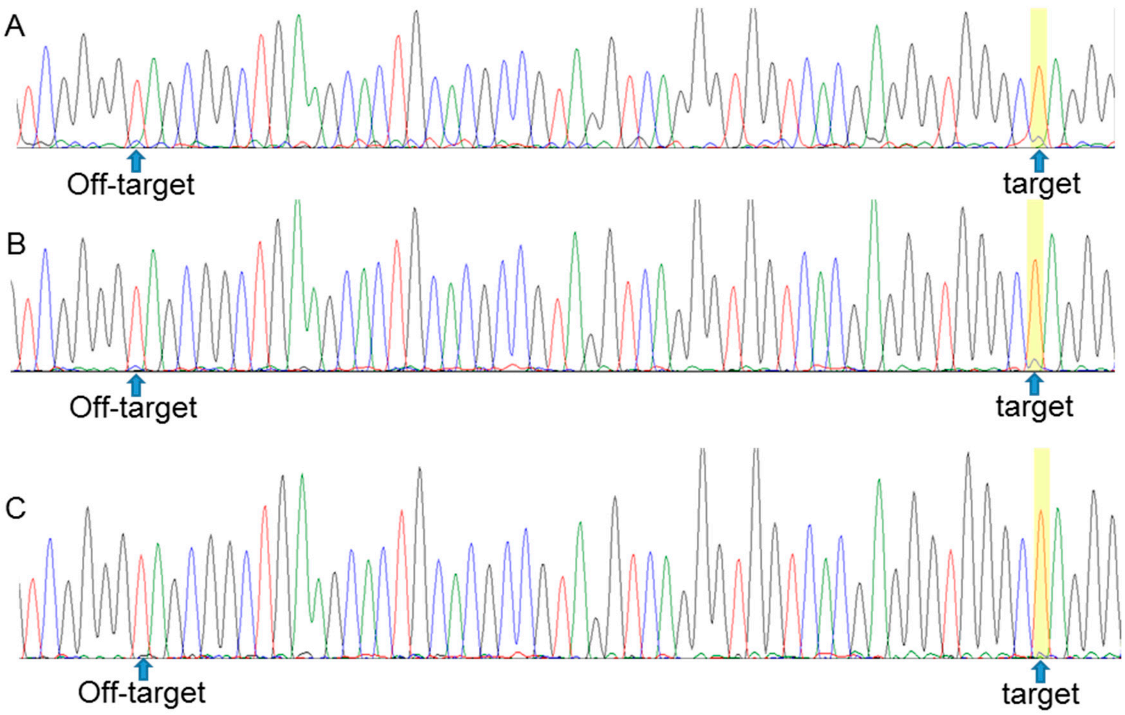

Supporting Figure S4. Sequencing analysis of *EGFP* in cells transfected with 500 ng of MS2-ADADR1-DD and 250 ng (**A**), 500 ng (**B**), or 1000 ng (**C**) of 21 nt upstream guide RNA.

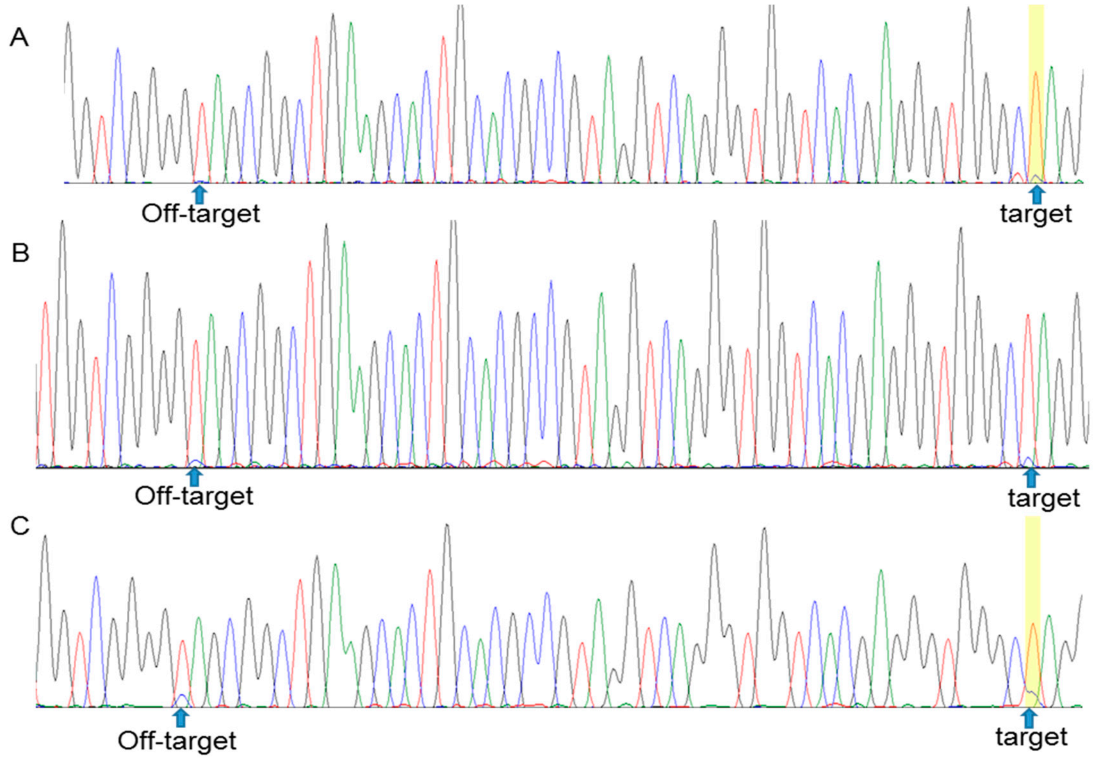

Supporting Figure S5. Sanger sequencing analysis of *EGFP* in cells transfected with 500 ng of double repeated 19 nt 2× upstream guide RNA and 250 ng (**A**), 500 ng (**B**), or 1000 ng (**C**) of MS2-ADAR1-DD.

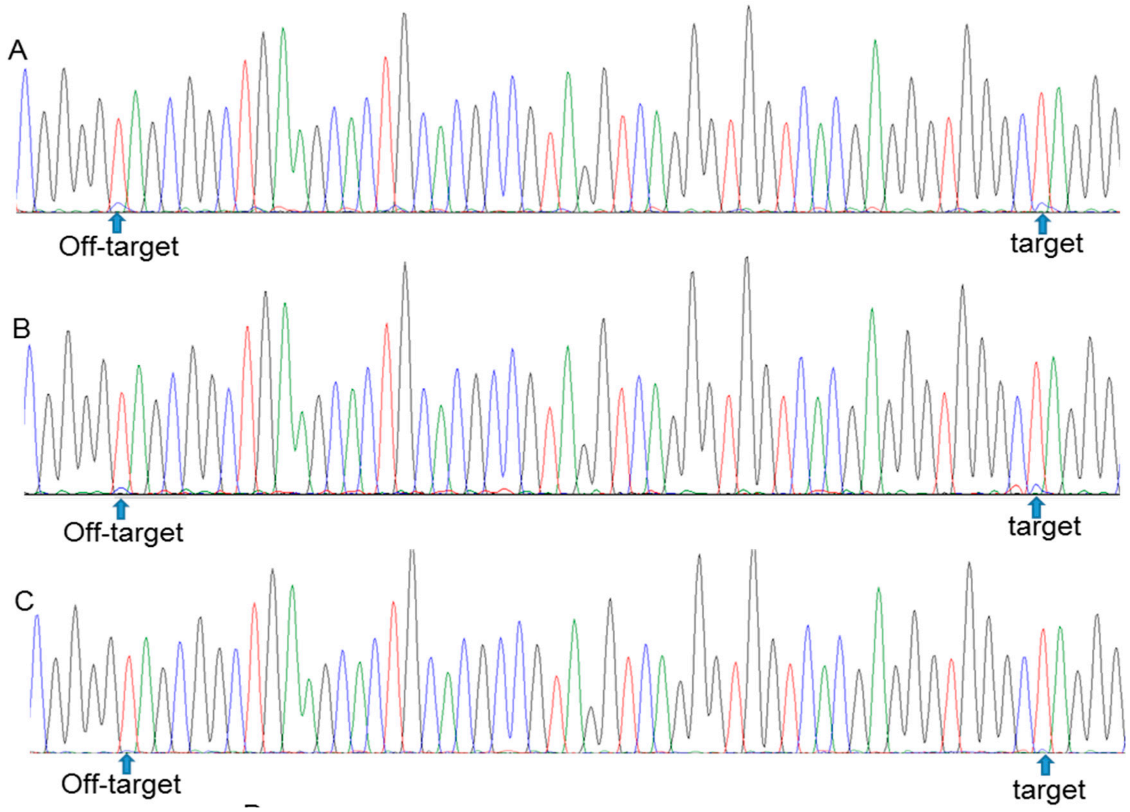

Supporting Figure S6. Sequencing analysis of *EGFP* in cells transfected with 500 ng of MS2-ADADR1-DD and 250 ng (**A**), 500 ng (**B**), or 1000 ng (**C**) of double repeated 19 nt 2× upstream guide RNA.

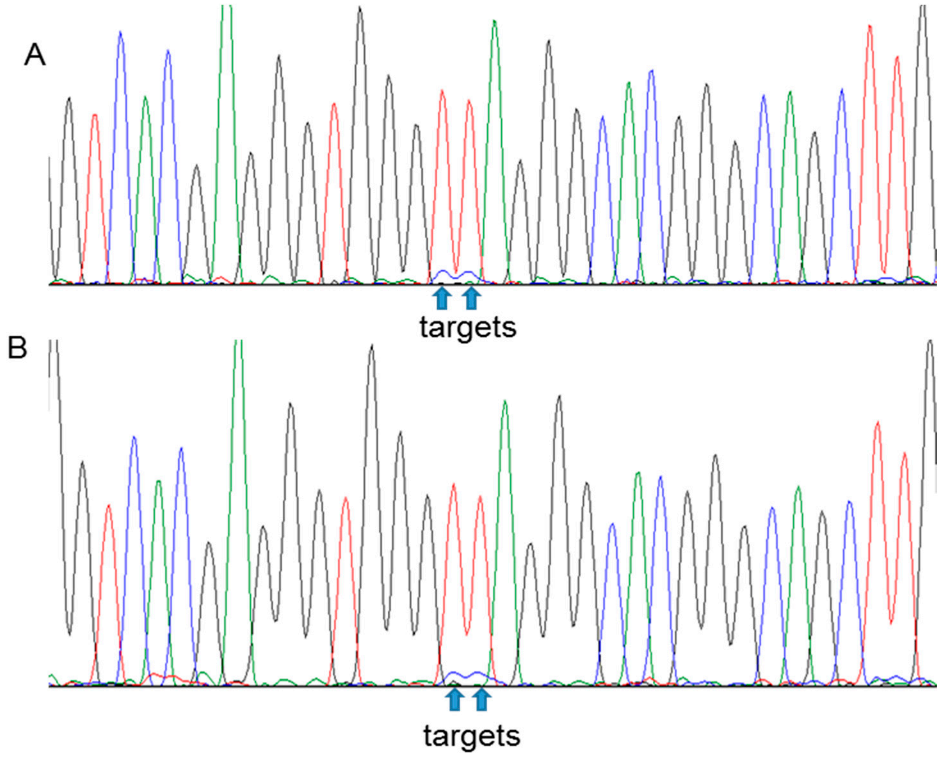

Supporting Figure S7. Sequencing analysis of the conversion of the ochre stop codon (TAA) to the tryptophan codon (TGG). (**A**) and (**B**) are replicates of the experiment. HEK-293 cells were transfected with 500 ng of MS2-ADADR1-DD and 250 ng of the 21 nt upstream guide RNA.

Bright image, left panel Fluorescence image, Right panel

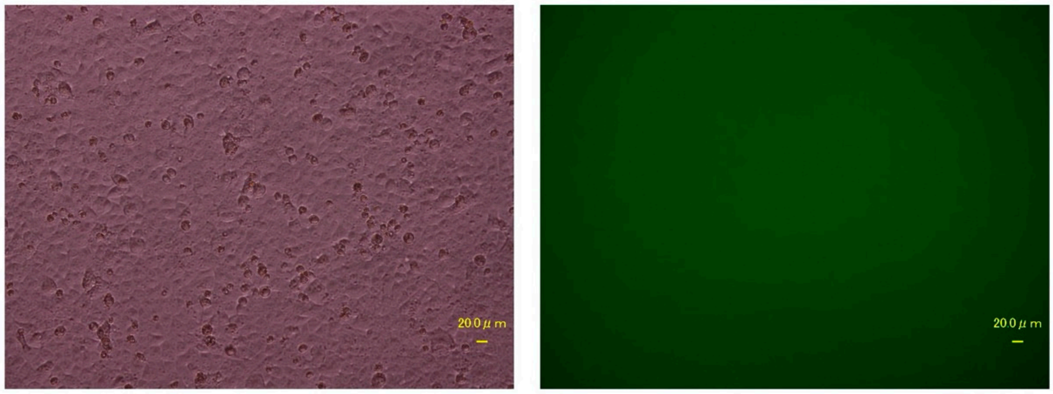

Fig. Negative Control

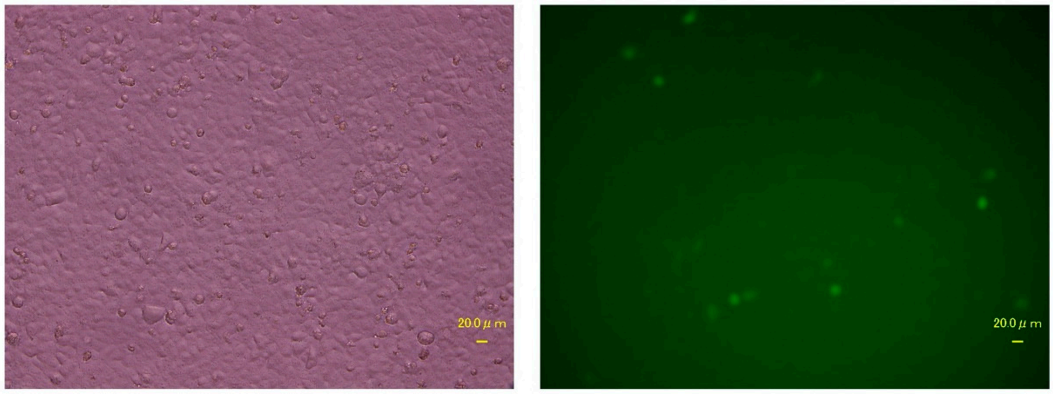

Fig. Experimental, MS2-ADAR1-DD, mutated EGFP and Guide RNA

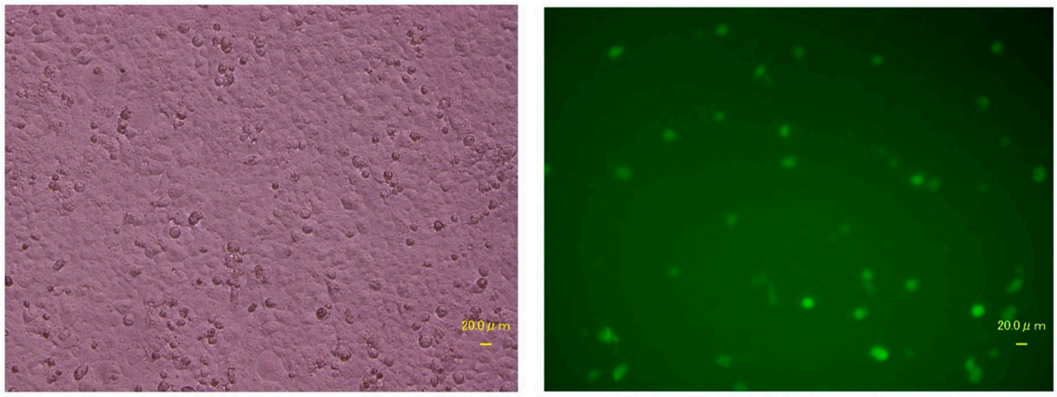

Fig. Positive Control, EGFP

Supporting Figure S8. Transfection experiment in HeLa cells. Bright image in left panel and fluorescence image in right panel of same focus. Figures were taken by Keyence Biozero fluorescence microscope, BZ-X800. Except the green fluorescence signal, the microscopic images show no remarkable observable differences among the treatment and control groups of cells
